# *DANSR*: A Tool for the Detection of Annotated and Novel Small RNAs

**DOI:** 10.3390/ncrna8010009

**Published:** 2022-01-13

**Authors:** Jin Zhang, Abdallah M. Eteleeb, Emily B. Rozycki, Matthew J. Inkman, Amy Ly, Russell E. Scharf, Kay Jayachandran, Bradley A. Krasnick, Thomas Mazur, Nicole M. White, Ryan C. Fields, Christopher A. Maher

**Affiliations:** 1 Department of Radiation Oncology, Washington University School of Medicine, St. Louis, MO 63108, USA; jin.zhang@wustl.edu (J.Z.); m.inkman@wustl.edu (M.J.I.); kayj@wustl.edu (K.J.); tmazur@wustl.edu (T.M.); 2 Institute for Informatics (I2), Washington University School of Medicine, St. Louis, MO 63110, USA; 3 Alvin J. Siteman Cancer Center, Washington University School of Medicine, St. Louis, MO 63110, USA; nmmaher@wustl.edu (N.M.W.); rcfields@wustl.edu (R.C.F.); 4 Department of Psychiatry, Washington University School of Medicine, St. Louis, MO 63110, USA; eteleeb@wustl.edu; 5 Department of Internal Medicine, Washington University School of Medicine, St. Louis, MO 63110, USA; erozycki@wustl.edu (E.B.R.); awilliam@wustl.edu (A.L.); 6 McDonnell Genome Institute, Washington University School of Medicine, St. Louis, MO 63108, USA; rscharf@wustl.edu; 7 Department of Computer Science & Engineering, Washington University, St. Louis, MO 63130, USA; 8 Department of Surgery, Washington University School of Medicine, St. Louis, MO 63110, USA; krasnickb@wustl.edu; 9 Department of Biomedical Engineering, Washington University, St. Louis, MO 63105, USA

**Keywords:** small noncoding RNA, colorectal cancer, next generation sequencing, bioinformatics software

## Abstract

Existing small noncoding RNA analysis tools are optimized for processing short sequencing reads (17–35 nucleotides) to monitor microRNA expression. However, these strategies under-represent many biologically relevant classes of small noncoding RNAs in the 36–200 nucleotides length range (tRNAs, snoRNAs, etc.). To address this, we developed DANSR, a tool for the detection of annotated and novel small RNAs using sequencing reads with variable lengths (ranging from 17–200 nt). While DANSR is broadly applicable to any small RNA dataset, we applied it to a cohort of matched normal, primary, and distant metastatic colorectal cancer specimens to demonstrate its ability to quantify annotated small RNAs, discover novel genes, and calculate differential expression. DANSR is available as an open source tool.

## 1. Background

A diverse range of small noncoding RNA (small ncRNA, 17–200 nucleotides) species have been shown to contribute to human development and diseases [1,2,3]. However, many existing large-scale research efforts, such as The Cancer Genome Atlas (TCGA), have generated data sets that enrich for small RNAs with lengths less than 35 nucleotides (nt) [4], which resulted in many small RNA species greater than 36 nt being under-represented. While this led to the development of tools that dramatically advanced the microRNA field [5,6,7], much remains to be understood about the biology of RNA species within the 36–200 nt range (Figure S1). To address this, we have optimized a method to enrich and sequence reads of 17 to 200 nt to capture the full diversity of small RNA species. This method enabled us to discover previously unannotated and differentially expressed RNAs in acute myeloid leukemia (AML) [8].

The ability to sequence variable read lengths can provide important insights concerning a class of small noncoding RNAs, as exemplified by tRNA fragments of varying lengths [9]. However, we were unable to apply the majority of existing small RNA analysis tools to our deep-sequenced data due to their inability to analyze sequence reads of varying lengths, inability to process larger quantities of sequence reads, or their lack of support for diverse small RNA species. A few tools capable of processing such data struggle with false positives and accurate classification of small RNA transcripts (Table S1). To overcome these limitations, we developed a new tool named DANSR, which can be broadly applied to large-scale sequencing data with variable read lengths to discover and quantify different classes of small noncoding RNAs.

## 2. Results and Discussion

### 2.1. Overview of DANSR (Detection of Annotated and Novel Small RNAs)

**Figure 1:**
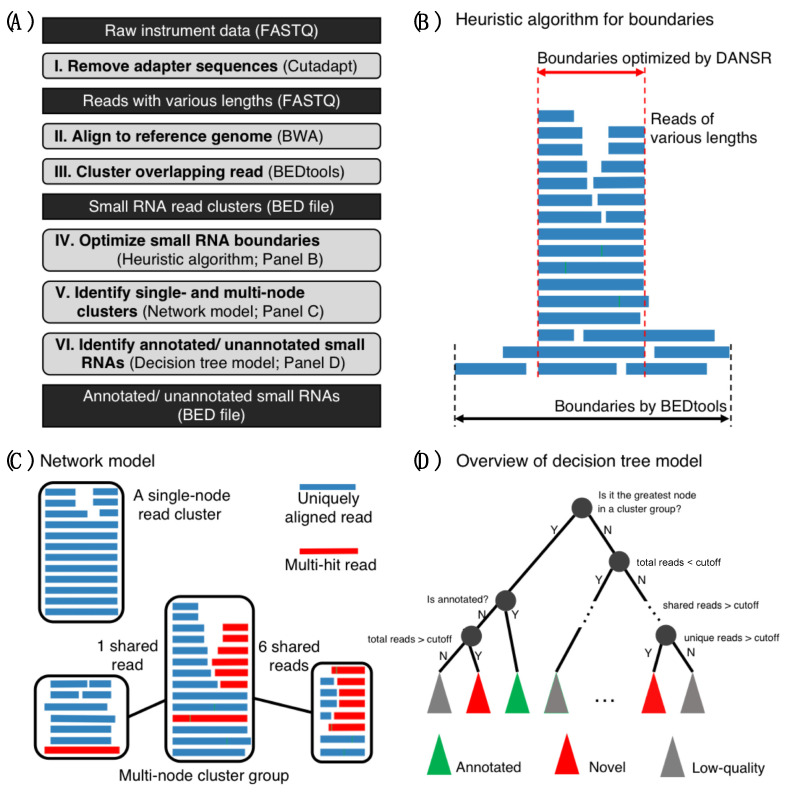
Optimized small noncoding RNA discovery and quantification tool, DANSR. (**A**) Workflow of the DANSR tool. DANSR consists of six steps for identifying and estimating small noncoding RNA expression. DANSR does not have any limitations on read length or size of small noncoding RNAs. (**B**) Improvement in small noncoding RNA boundary estimation. Sequencing reads are assigned a weight based on support from overlapping reads and low-weight reads are excluded to improve small noncoding RNA boundary discovery. (**C**) Network analysis of read clusters. A network is used to identify single-node read clusters and multi-node read clusters (group of clusters). (**D**) Overview of decision tree model to analyze read cluster networks (see Figure S3 for full detail). Low-quality, false positive read clusters are filtered and annotated and novel small noncoding RNAs are discovered and categorized.

To accurately discover and quantify small RNA (17–200 nt) expression, the DANSR tool executes six steps (Figure 1A): (1) Cutadapt [10] is used to remove the adapter sequences from the small RNA sequencing data in FASTQ/FASTA format, retaining reads of at least 17 nt in length. (2) BWA [11] is used to align sequencing reads and create output in SAM/BAM format, using parameters as previously described [8] to keep both uniquely aligned and multi-mapped reads. (3) The SAM/BAM file produced by BWA is converted to BED format and overlapping alignments are merged into read clusters using BEDTools [12]. (4) A heuristic algorithm (see the Section 4 below) is applied to optimize the boundaries of each read cluster to reconstruct the small RNA (Figure 1B). (5) A network is built based on uniquely aligned and multi-mapped reads to identify single-node read clusters and multi-node read clusters (i.e., a group of read clusters linked by shared multi-mapped reads) (Figure 1C), which are used in the next step to identify low quality read clusters caused by repetitive reads. (6) A decision tree model is applied to identify annotated and novel small RNAs (Figure 1D), comparing read clusters with a collection of annotated RNA species: microRNAs (miRNA) [13], piwi-interacting RNAs (piRNA) [14], transfer RNAs (tRNA) [15], small nucleolar RNAs (snoRNA) [16], small nuclear RNAs (snRNA) [17], ribosomal RNAs (rRNA) [18], and miscellaneous ncRNAs (miscRNA) [19]. Details of Steps (4)–(6) can be found in the Section 4 below.

The DANSR tool was implemented in the Python and C++ programming languages. A Docker image of the tool has also been provided to increase ease of deployment. Compiled small noncoding RNA annotations in GTF format, together with detailed instructions and examples for using the DANSR tool, are freely available on DANSR’s github page (https://github.com/ChrisMaherLab/DANSR, accessed on 25 October 2021). The input of DANSR includes sequencing reads from a sample in FASTQ/FASTA format and annotated small noncoding RNAs in GTF format (the DANSR website provides a comprehensive and up-to-date GRCh38 ncRNA annotation set curated by RNAcentral [20] and a hand-curated set of hg19 ncRNA annotations [8]; users may also provide their own small RNA annotation file). The output of DANSR includes tables of previously annotated small RNAs, RNAs in close proximity to annotated RNAs, and previously unannotated small RNAs. Results are reported in BED-compliant TSV format and include the genomic locus of each small RNA, the total number of supporting reads, the number of unique reads, and the number of multi-mapped reads. Annotated small RNAs also include the best annotation for the feature, its Jaccard score, and other possible annotations with lower scores.

### 2.2. Discovery of Dysregulated and Novel Small RNA Expression in Colon Cancer Progression

**Figure 2:**
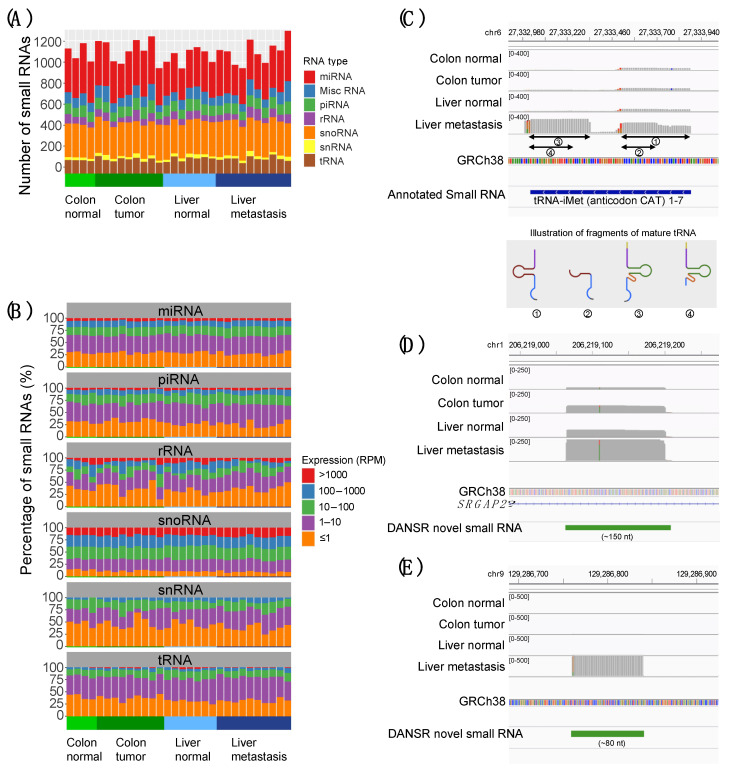
Expression of annotated and novel small noncoding RNAs in primary and metastatic colorectal cancer samples. (**A**) Number of annotated small RNAs identified in matched colon and liver samples for multiple small RNA species. (**B**) Expression of annotated small RNAs in matched colon and liver samples. (**C**) An example of tRNA expression (tRNA-iMet (anticodon CAT) 1–7). This previously annotated tRNA is shown to be supported by reads of different lengths that cover multiple fragments of the mature tRNA. (**D**) Example of a previously unannotated small noncoding RNA discovered by DANSR. This small RNA is intronic of SRGAP2, ~150 nt in length. (**E**) Example of another previously unannotated small noncoding RNA discovered by DANSR, which is intergenic and ~80 nt in length. The full list of previously annotated and unannotated small noncoding RNAs from the colorectal cohort is included in Table S3.

To demonstrate how DANSR can be used to discover small noncoding RNAs and dysregulated gene expression, we generated small RNA expression data from 9 primary colorectal tumor samples (4 with matched adjacent normal samples) and 10 distant liver metastases with matched adjacent normal liver tissue (Table S2) using a deep sequencing protocol similar to the one we designed and applied previously [8] (see the Section 4 below). We will refer to this dataset as the deep sequencing CRC cohort in this study. Altogether, ~3800 annotated small noncoding RNAs were identified by DANSR (Table S3A), and we observed expression of RNAs from each class of small noncoding RNAs (Figure 2A) across a wide range of expression levels (Figure 2B). DANSR’s ability to process reads of various lengths allows it to detect transcription of the full range of small ncRNA types. As one example of this application, Figure 2C highlights DANSR’s detection and correct annotation of multiple different tRNA fragments derived from a single annotated tRNA (tRNA-iMet (anticodon CAT) 1–7). The various fragments of the mature tRNA present in each tissue type are highlighted: while the full length of the tRNA is transcribed in liver metastatic tissue, fragments (2) and (3) have the most significant expression; fragments (1) and (4) have a lower level of transcription in normal colon tissue, while lower levels of fragments (1) and (3) are present in colon tumor and liver normal tissue. The role of tRNA fragments in cancer biology is an area of ongoing research interest [9,21], and Figure 2C points toward the potential utility of the DANSR algorithm in such studies.

We also used DANSR to discover >2900 previously unannotated small RNAs (Table S3C). To exemplify this, Figure 2D shows a previously unannotated intronic small RNA of ~150 nt expressed at both the colon and liver sites. Figure 2E shows another previously unannotated small RNA discovered by DANSR that is intergenic and ~80 nt in length.

Altogether, of the more than 7700 small RNAs (both the above annotated and unannotated RNAs as well as >1000 in close proximity to known annotations) detected by DANSR within the CRC cohort, 60.9% were intronic, 29.8% were intergenic, and 9.3% overlapped known exons of protein coding genes or long noncoding RNAs (lncRNA). Examples of DANSR’s successful detection of annotated small RNAs overlapping protein coding exons are illustrated in Figure S4. The focus of DANSR on small ncRNA detection via optimized boundary estimation and high accuracy RNA discovery in the network and decision tree modules allows it to sensitively identify annotated and novel small RNA clusters that overlap with such exons without cluttering the results with large numbers of RNAs representing highly-expressed protein coding genes and lncRNAs.

We also took advantage of the distinctive, hairpin-loop structure exhibited by miRNAs to sift through the set of unannotated small ncRNA gene candidates to identify novel miRNAs. RNAfold 2.4.14 was used to predict the secondary structure of the candidate genes from their corresponding DNA sequences [22]. These sequences contained the stem identified by DANSR, along with up to 40 additional nucleotides extracted in the direction of the remaining portion of the miRNA, including the loop and remaining stem, thereby forming a complete hairpin. Examples of DANSR’s novel miRNAs discovered in this fashion are illustrated in Figure S5.

**Figure 3:**
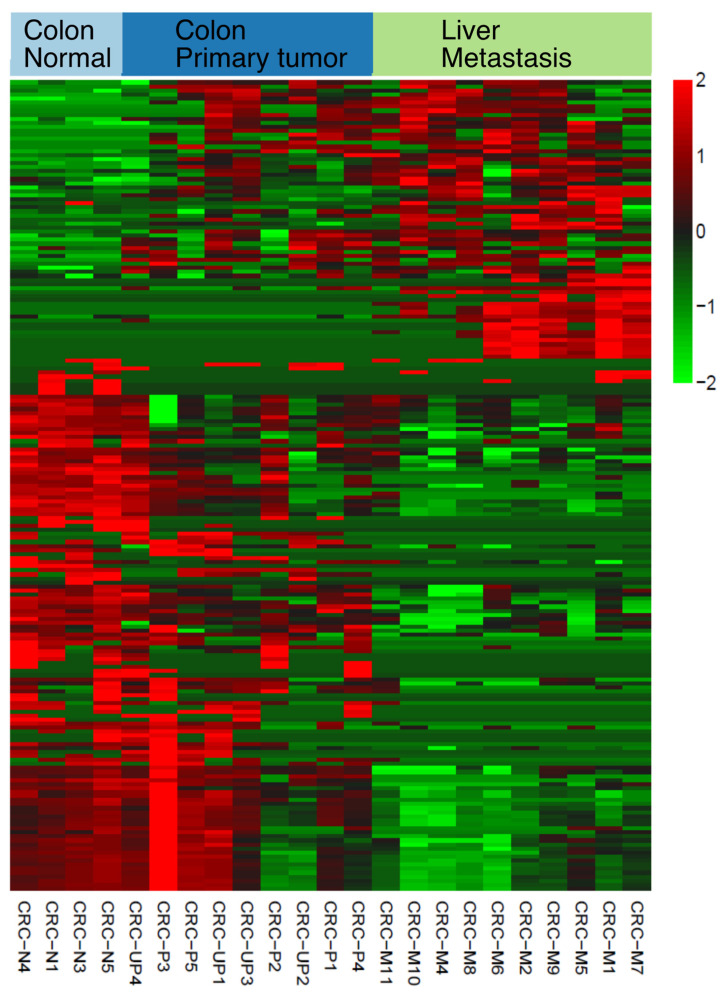
Differentially expressed (DE) small noncoding RNAs in colorectal cancer. Pairwise comparisons were performed for primary colon cancer, metastasis cancer, and adjacent primary colon samples (Table S4). The heatmap shows 204 DE small RNAs detected in at least two comparisons. Fold changes, significance levels, and expression levels can also be found in Table S4. N stands for normal colon, P stands for primary colon, UP stands for unpaired primary colon (no adjacent normal sample collected), and M stands for metastasis at liver. Normal liver samples were used to filter tissue specific DE genes between the two organs.

Using all previously annotated and novel small noncoding RNAs, we discovered differentially expressed (DE) RNAs in colorectal cancer as shown in Figure 3. Fold change and p-value were calculated using EdgeR [23] and included in Table S4, together with lengths and RPM values. Taken together, the results of this analysis demonstrate that DANSR is a tool optimized to discover, identify, and measure small noncoding RNA expression.

### 2.3. Comparison of Strategies for Utilizing Multi-Mapped Reads

**Figure 4:**
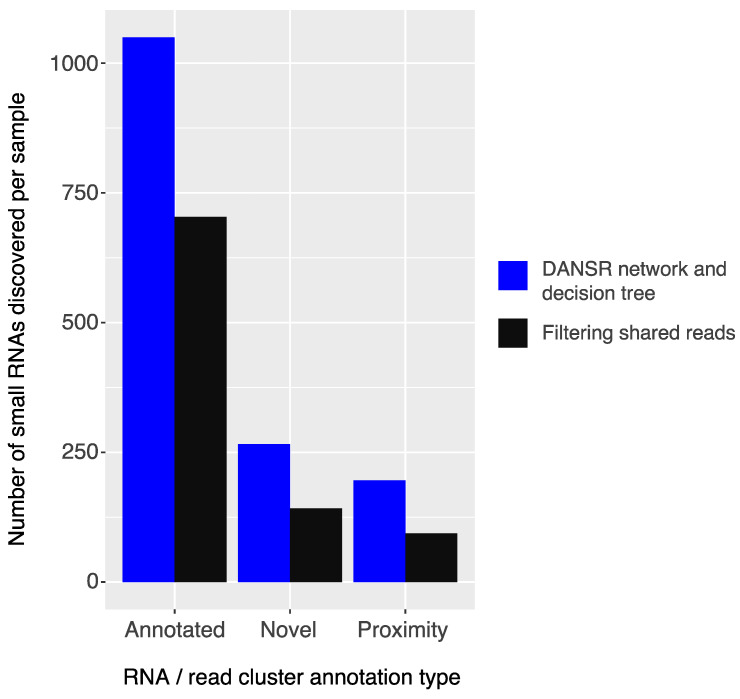
Comparison of approaches to multi-mapped reads. Average number of small RNA clusters discovered per sample in the deep-sequenced colorectal cancer cohort when using DANSR’s combined network model and decision tree approach to deducing the correct alignment for multi-mapped reads versus filtering shared reads. Clusters are grouped by annotation type: annotated small RNAs, novel clusters that are unannotated, and clusters in close proximity to a known annotation. DANSR’s approach results in significant numbers of small RNAs spanning homologous genomic regions remaining discoverable compared to the filtering approach.

To filter false positive clusters caused by multi-mapped reads, DANSR uses its network and decision tree models to identify the original loci of small ncRNA reads that map to large numbers of homologous regions across the genome. To evaluate our claim that the approach used by DANSR to analyze multi-mapped reads allows for more sensitive and accurate results, we compared DANSR with a naïve approach that simply removes all multi-mapped reads shared between nodes of the network graph from consideration, assuming they cannot contribute meaningfully to the discovery of small RNA clusters. Figure 4 presents the results of applying both approaches to the primary tumor samples of our deep-sequenced CRC cohort. Filtering shared reads reduced the average number of discoverable annotated small RNAs per sample by nearly a third and the numbers of novel clusters and clusters in close proximity to known small RNAs by nearly half when compared to DANSR’s decision tree approach. Given the highly homologous structure of many small RNA genes throughout the genome, the sensitivity of any tool that fails to account for such reads will be severely restricted. On the other hand, the specificity of any tool that retains such reads without a robust method of allocating multi-mapped reads to their true cluster of origin will be greatly attenuated, resulting in large numbers of false positive results.

### 2.4. Benchmarking Using Contemporary State-of-the-Art Tools

In order to assess DANSR’s performance, we sought to benchmark against a wide array of existing small RNA discovery tools. The majority of such tools proved to be unusable for our current application due to input file size, read length, or small RNA type restrictions (e.g., see Table S1). Two contemporary tools that were amenable to our deep-sequenced input are the widely applied and well-established ShortStack [24] and the recently developed Manatee [25]. Compared to the output of these tools, DANSR supplies users with complete information to characterize and prioritize discovered small RNAs: every candidate cluster is reported with genomic range, strand, total number of mapped reads, number of uniquely mapped reads, number of reads shared with other clusters, and annotation group (annotated, unannotated, or low quality); annotated clusters additionally report the name and biotype of all candidate features, the categories of candidate features (small RNA, protein coding gene, pseudogene, and lncRNA), the best feature, and its associated Jaccard score. In contrast, ShortStack supplies only the genomic ranges of clusters but does not provide any corresponding annotation; Manatee does not supply the numbers of uniquely mapped and shared reads for unannotated clusters, and for annotated clusters does not report the genomic coordinates of the cluster. To facilitate comparisons of ShortStack’s performance in small RNA detection with the other two tools, we annotated its reported clusters using DANSR’s own Jaccard score-based annotation function.

**Figure 5:**
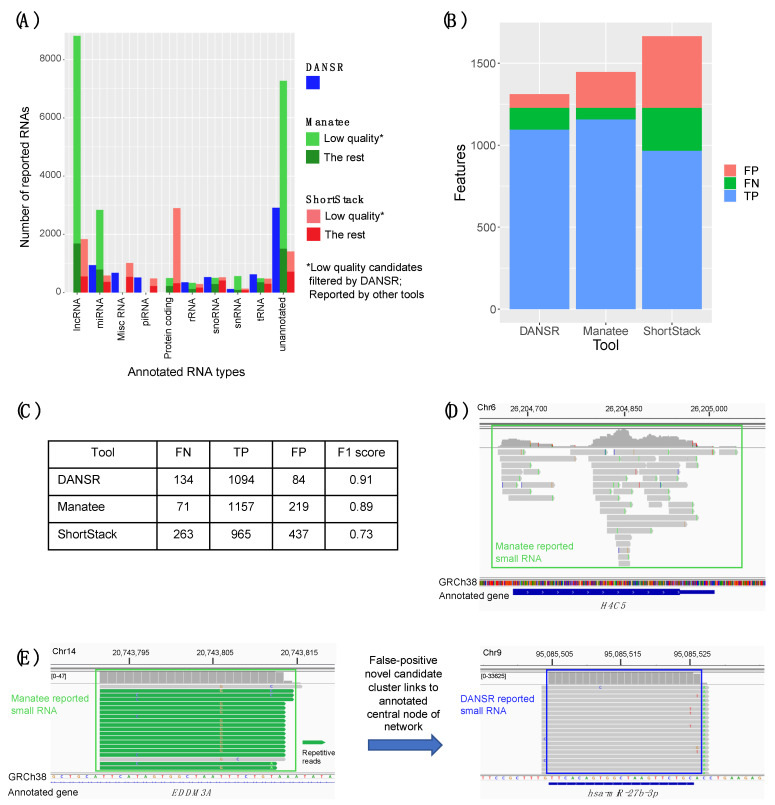
Benchmarking using contemporary small RNA discovery tools. (**A**) Number of unique clusters of each small RNA species and novel, unannotated clusters identified within the primary colon tumor samples of the deep-sequenced CRC cohort by DANSR, Manatee, and ShortStack. Annotated and novel clusters discovered by DANSR are plotted in dark blue; clusters discovered by Manatee that were not rejected by DANSR are plotted in dark green, while clusters reported by Manatee that were rejected by DANSR for failing to pass quality filters (clusters of repetitive reads containing no reads with a unique mapping, clusters that share most reads with a better quality cluster at another genomic location, clusters representing expression of protein coding genes, etc.) are plotted in light green; clusters discovered by ShortStack that were not rejected by DANSR are plotted in dark red, clusters that failed DANSR’s quality filters in light red. (**B**) Benchmarking results for each tool on a set of simulated ncRNA read data; true positives (TP) are in blue, false negatives (FN) in green, and false positives (FP) in red. (**C**) DANSR demonstrates the best overall performance in detecting simulated small RNAs as determined by F1 score. A false negative is a case in which a small RNA with simulated transcript count >5 was estimated as 0 by a tool; a false positive is a case in which a tool estimates transcript counts >5 for which the true value was 0. (**D**) Manatee reports as novel small RNA discoveries over 1900 clusters that DANSR correctly identifies and rejects as expression from protein coding genes, an example of which is seen here. (**E**) Manatee reports as results over 15,000 clusters that fail to pass DANSR’s QC filters, including over 400 with significant read support, as seen here; the cluster on the left is reported by Manatee as a novel small RNA, while DANSR’s network model for shared multi-mapped reads and decision tree model are able to determine this cluster as a false positive from reads that map to the much more highly expressed, annotated cluster on the right.

Another advantage of DANSR is its ability to distinguish true small RNA read clusters from false positives that arise from reads that align to multiple locations and from protein-coding gene expression. Figure 5A shows the number of annotated and novel RNAs reported in the nine primary tumor samples of our institutional deep-sequenced CRC cohort by DANSR, Manatee, and ShortStack. Among the three tools, we noted that Manatee reports a large number of candidate small RNAs that are actually from annotated lncRNAs (N=8814). Among small ncRNA types, DANSR reported slightly more (piRNA, rRNA, snoRNA, tRNA) or a much smaller number of candidates (miRNA, snRNA, unannotated) compared to the other tools. For example, DANSR reported 947 unique miRNAs among these samples, while Manatee reported 2844 and ShortStack 587.

We suspected that DANSR’s reporting fewer small RNAs among several types was due to its network and decision tree models successfully filtering false positive candidates. To test this, we compared the reported results from the competing tools to DANSR’s tables of rejected candidate small RNA clusters, i.e., clusters identified as low-quality, false positives by the DANSR algorithm. We observed that over 15,000 and 5900 clusters rejected by DANSR are reported as true results by Manatee and ShortStack, respectively. When filtering these rejected clusters from the results, DANSR consistently reported larger numbers of high-quality small ncRNAs in all the small RNA species. For example, after removing the rejected miRNA candidates, only 795 miRNA reported by Manatee were retained and only 368 miRNA reported by ShortStack were retained, compared to 947 annotated miRNAs reported by DANSR. We manually inspected the low-quality, false positive clusters rejected by DANSR but reported by Manatee and ShortStack. Figure 5D,E show two representative cases of such read clusters that were rejected by DANSR but reported by Manatee as small RNAs. In Figure 5D, transcripts from the deep sequencing data of a one-exon protein-coding gene, *H4C5*, were reported as a novel small RNA, one of over 1900 such clusters rejected by DANSR as transcription of protein coding or pseudogenes. On the left, Figure 5E shows one of over 400 ambiguous read clusters with significant read support reported by Manatee as novel small RNAs that DANSR’s network model for shared multi-mapped reads and decision tree model identify as a false positive. This cluster is made up of reads that also map to the much more highly expressed and annotated cluster on the right, one much more likely to represent a true small ncRNA.

To further assess the performance of each small ncRNA discovery tool against a known benchmark, all three tools were run on the set of simulated small RNA reads created by the authors of Manatee (simulated_sRNA_MonteCarlo.fa) and the matching ncRNA annotation file (ncRNA_hg38.gtf). The tags of the GTF file were appended to include the transcript_id field expected by DANSR. To facilitate a fair comparison, lncRNA and protein coding genes, which are automatically filtered from DANSR’s results, were removed from the set of input reads, and clique graphs from DANSR’s network model, where a single group of reads is identified as aligning to a group of repetitive regions, were designated as a true positive (TP) if the group contained the origin of the simulated reads. As in the Manatee paper, a false negative (FN) was defined as a case in which a small RNA with simulated transcript count >5 was estimated as 0 by a tool; a false positive (FP) was a case in which a tool estimated transcript counts >5 for a small RNA whose true value was 0. Figure 5B,C present the results of this test. DANSR had the best overall performance on the simulated data, detecting a roughly equal number of TP clusters as Manatee (1094 vs. 1157) with fewer FP clusters (84 vs. 219). The results of this simulation reinforce the picture obtained from the test on the real data from our deep CRC cohort, that DANSR has equal or greater sensitivity to true small ncRNA expression when compared with other tools, while providing better control of false positives.

### 2.5. Estimating Boundaries in Mid-Sized Small RNA Discovery

**Figure 6:**
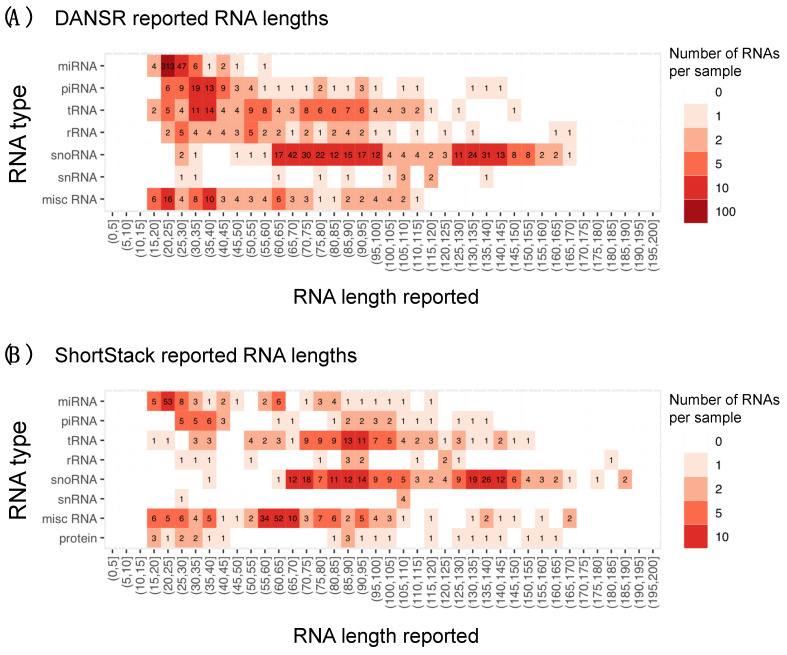
Length distribution of expressed annotated small RNA read clusters. The advantage of DANSR’s boundary optimization algorithm can be seen by comparing the length distribution of expressed annotated small RNAs identified by DANSR in the primary colon tumors to those identified by ShortStack. The expressed and annotated small RNAs are binned by length to different length ranges up to 200 nt. Each bin differs by 5 nt. Darker colors indicate that a higher average number of annotated small RNAs are expressed per sample in this range. (**A**) In DANSR’s results, 85% of miRNAs are concentrated in the 17–25 nt range, 60% of piRNAs are in the 20–40 nt range, and 93% of snoRNAs fall between 60 and 150 nt, as would be expected from the known structure of these small RNA species. (**B**) ShortStack, lacking a cluster boundary optimization step, reports expression of many features well outside their known length ranges; for example, 40% of miRNAs have lengths above 25 nt and 57% of piRNAs have lengths above 40 nt.

To accurately build the network to handle multi-mapped reads and apply the decision tree model to classify RNA species, it is critical to accurately estimate the boundaries and lengths of the small RNA candidates. This issue was less critical when the focus was on the length range of 17–35 nt, where the longest small RNA is only about twice the size of the shortest RNA. However, for the full length range of 17–200 nt, the longest RNA can be ~12 times longer than the shortest RNA. The advantage of DANSR’s boundary optimization algorithm can be seen by comparing the length distribution of expressed annotated small RNAs identified by DANSR in the primary colon tumors to those identified by ShortStack (Figure 6) (Manatee cannot be included in this comparison as it does not report length ranges of annotated clusters). The cluster boundary optimization algorithm within DANSR ensures that annotated clusters correspond closely to the expected length ranges of their assigned small RNA type: e.g., 85% of miRNAs were concentrated in the 17–25 nt range, 60% of piRNAs were in the 20–40 nt range, and 93% of snoRNAs fell between 60 and 150 nt (Figure 6A). In contrast, ShortStack lacked boundary optimization, resulting in significant numbers of read clusters that fell far outside the known range of their assigned species type or group. This lack of optimization also produced multiple clusters that should in fact represent more than one distinct small RNA. As an example, for annotated miRNAs that are expected to be in the 17–25 nt length range, 40% of ShortStack-identified miRNAs were reported with lengths greater than 25 nt, while 57% of piRNAs fell outside of the expected 20–40 nt length range (Figure 6B). The greater correspondence between clusters of known small RNA reads and their reference annotation is additionally illustrated in Figure S2B–D, which presents the improvements in Jaccard scores across all samples of the CRC cohort for miRNAs, piRNAs, and snoRNAs, respectively.

### 2.6. Novel RNAs Confirmed Using TCGA Colon and Rectum Cancer Cohorts

**Figure 7:**
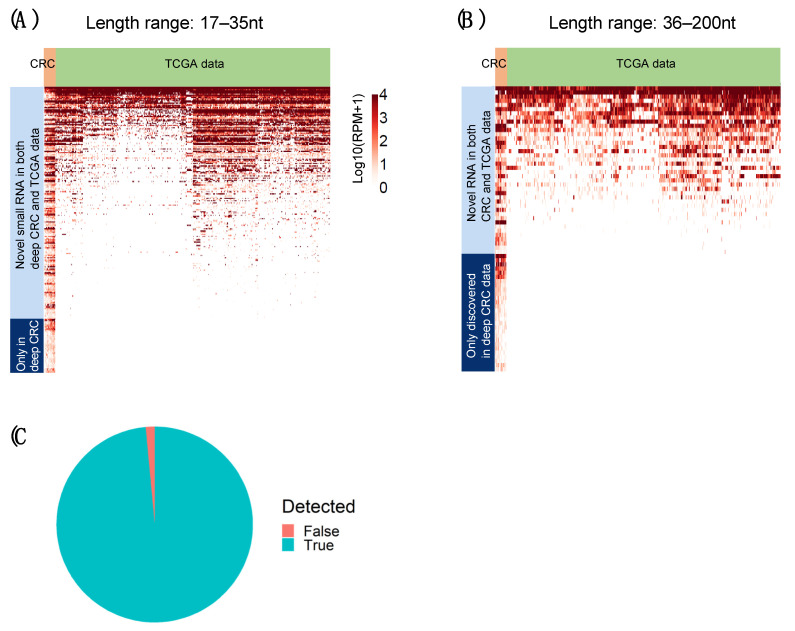
Expression of novel small RNA clusters detected by DANSR in the deep-sequenced CRC cohort among TCGA-COAD and TCGA-READ miRNA-seq samples. (**A**) A large majority of novel clusters at the short end of the length range (17–25 nt) are discoverable within the TCGA miRNA-seq samples (82%, or 207 of 255 novel small RNAs), and those that are present within the TCGA data tend to be expressed consistently across large numbers of samples. (**B**) Novel clusters at the longer end of the length range (36–200 nt) are still discovered within the TCGA miRNA-seq data, but due to the sequencing protocol’s focus on enrichment for miRNA in the 17–35 nt range, they are discovered at a lower rate (58%, or 40 of 68 novel small RNAs) and in a lower proportion of samples. This figure includes RNAs that were discovered in at least 50% of samples in the institutional CRC cohort. The full list of novel small ncRNAs is included in Table S3. (**C**) DANSR successfully detects 99% (466 out of 473) of miRNAs that TCGA reports as being expressed within either TCGA-COAD or TCGA-READ.

To assess the reliability of de novo detections of small RNAs by DANSR in our deep-sequenced CRC cohort, we used the miRNA data (enriched for 17–35 nt) from TCGA colon adenocarcinoma (COAD) and rectum adenocarcinoma (READ) cohorts as a validation cohort [4]. Figure 7A,B compares the expression of novel small RNAs commonly detected within the deep-sequenced CRC cohort (defined as detection within >50% of samples) between the deep-sequenced and TCGA miRNA sample set. Figure 7A demonstrates that a large majority of common novel RNAs on the shorter end of the range (17–35 nt) discovered by DANSR within the deep-sequenced CRC cohort (82%, or 207 of 255 novel small RNAs) were also found within TCGA colon and rectum miRNA-seq samples. Figure 7B shows that a lower proportion (58%, or 40 of 68 novel small RNAs) of longer novel small RNAs (36–200 nt) are discoverable within TCGA data. This difference is to be expected, as the library preparation of our institutional deep-sequenced cohort is designed deliberately to enhance sensitivity to include the full length range of small RNA species in the 17–200 nt length range, while the miRNA-seq library preparation utilized by TCGA only enriches for miRNA in the 17–35 nt length range. Nevertheless, the ability to detect the same novel small RNA clusters within both cohorts across the relevant length range confirms that DANSR is capable of successfully detecting novel small RNA species.

The TCGA miRNA enriched cohorts also provide an opportunity to independently benchmark DANSR’s performance in the discovery of annotated miRNAs. DANSR successfully detects 99% (466 out of 473) of miRNAs that TCGA reports as being expressed within either TCGA-COAD or TCGA-READ (defined as at least one sample with >5 RPM by TCGA), as shown in Figure 7C.

## 3. Discussion

Our study shows DANSR is a novel tool that represents a valuable addition to existing methods in the area of small ncRNA detection, quantification, and discovery. DANSR implements the algorithms that have been empirically established through our prior ncRNA discovery studies, making these powerful methods easily available to users [8]. Further, we have demonstrated improved performance of DANSR through its boundary optimization algorithm, multi-node cluster network model, and decision tree model for discovering and classifying candidate small RNA read clusters (Figure 1). The boundary optimization method used by DANSR greatly increases the Jaccard scores of known small RNA species in relation to their annotation (Figure S2B–D), increasing its ability to accurately annotate known small ncRNAs. It also produces accurate size estimates for known and novel small ncRNAs discovered among the RNA-seq data, facilitating classification and eventual functional analysis of these novel clusters (Figure 6A). Using a multi-node cluster network model allowed for accurate interpretation of the significant number of multi-mapped reads due to homology among small ncRNAs. False positive clusters consisting solely of multi-mapped reads that have alternate alignments within clusters containing uniquely aligned reads are rejected; the multi-mapped reads are instead assigned to the proper genomic locus of RNA expression with unique read support. In addition, small ncRNAs with multiple copies throughout the genome are counted only a single time when calculating expression levels. Finally, the application of a carefully calibrated decision tree model (Figure S3) to each cluster within a graph created by the network model ensures that low-quality read clusters are rejected, reducing false positives, while clusters of acceptable quality are accurately classified as annotated, in close proximity to known annotation, or unannotated small ncRNAs.

We have demonstrated the effectiveness of the DANSR model by applying it to a deep-sequenced CRC cohort consisting of colon tumor, matched normal, liver metastasis, and liver normal samples whose library preparation was designed specifically to facilitate the discovery of both short (17–35 nt) and mid-length (36–200 nt) ncRNAs (Figure 2). DANSR was able to detect over 7700 small ncRNA clusters within the deep-sequenced CRC cohort, including 204 small RNAs with significant differential expression in at least two of the pairwise comparisons between colon normal-colon tumor, colon tumor-liver metastasis, and colon normal-liver metastasis (Figure 3), thereby illustrating DANSR’s utility in exploring the role of ncRNAs in cancer biology. In addition, we highlighted one of many examples in which DANSR detected different fragments of a particular tRNA in different tissue types from a single patient (Figure 2C), demonstrating our tool’s potential contribution to another area of ongoing research interest. While we applied DANSR to a cancer related study in this paper, DANSR is broadly applicable to any small RNA dataset to study different phenotypes users are interested in.

We examined the results of a comparison between DANSR and two alternative tools, Manatee and ShortStack. In addition to DANSR’s more complete output, with full annotation and cluster range information, we saw that both tools returned many thousands of results that DANSR’s algorithm rejected as low-quality (Figure 5A). Manual inspection of such clusters confirms that they are false positives of a variety of types, including misreported expression of protein-coding exons and sub-optimal clusters of multi-aligned reads (Figure 5D,E). In contrast, Figure S4 demonstrates DANSR’s ability to identify true novel and annotated small ncRNAs that overlap protein coding and lncRNA genes without reporting large numbers of false positive results consisting of protein coding and lncRNA transcripts. Excluding such low-quality clusters, DANSR detects more small RNAs across all species types, as well as a roughly equal number of novel clusters, when compared with the other tools. This result highlights its superior performance in sensitively detecting true small RNA clusters while filtering false positives. The potential for novel small ncRNA discovery using DANSR is illustrated in Figure S5, which shows novel miRNAs with hairpin precursors identified using DANSR’s unannotated candidate clusters for deep-sequenced CRC samples. DANSR’s superior ability to sensitively detect true positive clusters while controlling false negatives was confirmed by simulated data (Figure 5B,C).

Finally, we used data from the TCGA-COAD and TCGA-READ studies as a positive control to benchmark DANSR’s ability to detect known and novel small ncRNAs. We ran DANSR directly on these data and detected, as expected, over 99% of the known miRNAs identified by TCGA (Figure 7C). This finding demonstrated that DANSR can be applied to existing miRNA data to report high quality results, while further benefiting from DANSR’s novel modules. Moreover, we used the TCGA miRNA-seq cohorts to validate the common novel small RNA species detected by DANSR within >50% of samples in the deep-sequenced CRC cohort. These analyses demonstrated the reliability of DANSR’s novel discoveries. A large majority (82%) of the novel small RNAs commonly discovered in the cohort at the short end of the length range (17–35 nt) were also detected in TCGA samples, indicating that these novel small RNAs are real and common across individuals with this cancer type (Figure 7A). The advantage of the greater sequencing depth applied to our CRC cohort is demonstrated by the additional 48 novel clusters present in >50% of samples in our cohort at lower levels of expression, which were not detectable among the TCGA miRNA-seq data. In the mid-size range of 36–200 nt, 42% of common novel small RNAs could not be detected within TCGA samples (Figure 7B). The presence of these novel clusters in a majority of deep-sequenced individuals creates confidence that such calls are true positives, again demonstrating the advantage of a deep-sequenced cohort optimized to facilitate discovery within the full 17–200 nt length range. These common mid-sized ncRNAs could have biological implications in these diseases, making the ability to detect them important.

## 4. Methods

### 4.1. Implementation of DANSR Tool

The DANSR tool is implemented in the Python and C++ programming languages and employs six main steps (Figure 1A): (1) adapter trimming, (2) read alignment, (3) cluster identification, (4) boundary optimization, (5) identification of single and multi-node clusters, and (6) identification of annotated and unannotated small RNAs. Each step in this pipeline and the default values of its associated parameters were implemented based on the lessons learned in overcoming the challenges to small RNA discovery in our previous study [8].

### 4.2. Standard Data Input/Output Format and Small RNA Annotation

In the optional first step, DANSR trims user-supplied adapter sequences from the submitted reads using Cutadapt [11], retaining reads of at least 17 nt (the minimum length of small RNAs). DANSR accepts small RNA sequencing data in the FASTQ/FASTA format.

The next step aligns the reads to the human reference genome using BWA ver. 0.7.17 [11]. The aligned reads are output in SAM/BAM format. Although alignment parameters can be defined by the user, use of the default values (-q 5 -k 1 -l 17), which were found to produce excellent results in earlier efforts to discover small noncoding RNAs [8], is highly recommended. 

In the third step, clusters are identified based on the overlap between read alignments, with several tunable quality filters applied to the reads before clustering. First, reads are filtered on their CIGAR string such that only insertions or deletions of lengths no more than 5 are allowed, and all indels must be flanked by alignments of lengths of at least 5 (these restrictions may be tuned using the parameter file cigars_allow.txt). Second, aligned reads are grouped into uniquely mapped reads, multi-mapped reads, and repeat reads. Unique and multi-mapped reads are retained, while repeat reads are excluded from further analysis; the number of alignments above which a read is designated as repeat and discarded is controlled by the number-hits parameter (default 5). To form clusters of reads, the retained reads are first converted into BED format and overlapped alignments are merged into read clusters using BEDTools (2.27.0 or later) [12]. Clusters containing fewer than the minimum allowable number of reads (defined by the number-reads parameter, default 5) are then excluded from further analysis.

### 4.3. Optimize Small RNA Boundaries

In the fourth step, a heuristic algorithm is employed to optimize the boundaries of each read cluster identified in the third step to more accurately define the range of the small RNA cluster and split it into sub-clusters if it represents more than one feature. (Figure 1B and Figure S2). This algorithm works by assigning a weight to each read based on the level of its contribution to the original cluster. The read weight is computed by first walking through the cluster bases and computing the read depth of each base (number of reads covering the base). An individual read’s weight is defined as the total coverage of the bases it spans divided by the read length. Thus, if p represents the position of the *i*th base in a read of length *n*, the read weight can be represented by the following equation:

(1) $$R_{w}=\frac{\sum_{1}^{n}cov\;\left(p_{i}\right)}{n}$$

where *cov* = total number of reads covering the base.

Once all read weights are computed, reads within the cluster are ordered by weight, and a number of the lowest-weight reads equal to a proportion of the total number of reads (defined by the percent-cur parameter, default 0.3) in the original cluster, designated $H_{0}$, are provisionally removed. The updated cluster ($H_{1}$) is then split into sub-clusters, each of which starts at a transition from 0 read coverage to coverage > 0 and ends at a transition from coverage > 0 to 0. $H_{0}$ is also subdivided into sections at the midpoint of each gap between sub-clusters in $H_{1}$. For each candidate sub-cluster, the average coverage depth is compared to that of the corresponding section of $H_{0}$. If the ratio of coverage depths $W(H_{0})/W\left(H_{1}\right)>\left(1+\mathrm{cutoff}\right)$, where cutoff is the input parameter (default 0.3), the reads and boundaries of the new sub-cluster in $H_{1}$ are accepted; otherwise, the initial cluster reads and boundary from the corresponding section of $H_{0}\;$are retained. Consecutive retained sections of $H_{0}$ are merged into single clusters. Figure S2 shows a sketch illustrating this process.

### 4.4. Identify Single- and Multi-Node Clusters

In the fifth step, a network model is built based on uniquely aligned and multi-mapped reads to identify single-node read clusters and multi-node read clusters (i.e., a group of clusters that share multiply-aligned reads between them) (Figure 1C), which are used in the next step to identify low quality clusters caused by repetitive reads. The network consists of a graph in which nodes represent clusters and edges represent the shared reads between clusters. Thus, if a cluster is comprised of only uniquely mapped reads, the cluster is called a single-node cluster. All single-node clusters are reported as results and do not need to be processed by the decision tree model in the sixth step. In its general construction, the network is a graph consisting of a large number of subnetworks. To assess the quality of the clusters, all multi-node clusters are subjected to a quality check in the next step.

Each node of the network is a cluster of read alignments, and two nodes are connected by a link if >50% of the alignments from at least one node have a hit in the other node. Fully connected sub-networks come in two shapes: star-like and clique. A star-like sub-network represents the case in which a central small RNA node generates false positive read clusters at multiple locations, which are difficult to filter in the absence of network analysis. A clique shape represents a small non-coding RNA that has multiple copies on the human genome. The network analysis ensures that, on the one hand, no copy of the same small non-coding RNA is missed and, on the other hand, that the expression level of the small non-coding RNA is calculated accurately by including reads from repetitive small RNAs only once.

At the same time as each cluster is processed by the network model, it is annotated using a human reference annotation and one or more small RNA annotation sets (all in GTF format). DANSR provides a GRCh38/hg38 small RNA library that includes annotations for >187 k features and is sourced from RNAcentral [20]. A curated GRCh37/hg19 small noncoding library collected from multiple resources by our team to cover any small RNA genes missing from the standard human genome annotation in a prior study [8] is also provided; users may employ either annotation file or provide their own. Clusters that overlap one or more features in the provided annotation files are provisionally annotated with each of those features.

### 4.5. Identify Annotated and Unannotated Small RNAs

To evaluate the quality of clusters and reduce the false positive rate, a decision tree model has been constructed to classify clusters (graph nodes) into three main categories: (1) annotated, (2) unannotated, and (3) low-quality clusters (Figure 1D). The decision tree model works by traversing each subgraph of the network and asking questions (Figure S3) based on the properties of each cluster to determine its class. The largest node in each cluster is classified as annotated if it overlaps one or more regions in the small RNA annotation file; otherwise, it is classified as unannotated or low-quality based on whether the number of reads within the cluster exceeds the cutoff value for acceptance (number-reads parameter, default 5). Each of the smaller nodes in a graph are then processed. First, a check is carried out to determine whether the number of reads within the cluster falls below the cutoff value for acceptance; if so, the cluster is classified as low quality. Then, the proportion of reads shared with the largest cluster of the graph is checked; if it exceeds a threshold (ov-with-largest parameter, default 0.75), the cluster is classified as low quality. Otherwise, the proportion of unique reads within the cluster is checked; if it exceeds the cutoff (percent-uniq parameter, default 0.5), the cluster is classified either as annotated (if it overlaps one or more annotations) or unannotated. Otherwise, the proportion of reads shared with other clusters is checked; if it is less than 50%, the cluster is classified either as annotated (if it overlaps annotations) or unannotated. Otherwise, the number of uniquely aligned reads within the cluster is counted; if it exceeds a cutoff value (unique-reads parameter, default 2), the cluster is classified either as annotated (if it overlaps annotations) or unannotated, and otherwise as low-quality.

Once all clusters are classified, low-quality clusters are written to the output file of rejected candidates and the remaining read clusters are further refined. Among annotated clusters (including single-node clusters that did not pass through the decision tree), if the highest Jaccard score among its provisional annotations is greater than a cutoff value (jaccard-index parameter, default 0.3), that annotation is applied to the cluster and it is written to the annotated output file. Clusters with scores below this value that fall within the length range 17–200 nt and that do not overlap with protein coding exons are designated as falling in close proximity to a known annotation and written to the close proximity output file; otherwise, the clusters are written to the rejected file. Clusters identified as unannotated are filtered for length and written to the unannotated output file.

### 4.6. Sequencing Protocol for Small RNAs in the 17–200 Nucleotide Range

In this paper’s application, we have improved the protocol [8] using Blue Pippin (Sage Science, Beverly, MA, USA) to enrich for library fragments in the full length range of 17–200 nt. Total RNA was isolated from snap-frozen tissue samples using TRIzol™ Reagent (ThermoFisher Scientific, Waltham, MA, USA). Working on dry ice, 2–5 mm Tissue Lyser beads (QIAGEN, Hilden, Germany) and 1.0 mL of TRIzol™ Reagent were added to a 100 mg tissue sample in a 2.0 mL Safe-lock Tube (Eppendorf, Hamburg, Germany). The sample was homogenized using the Tissue LyserLT (QIAGEN, Hilden, Germany) at top speed for 10 min at room temperature. For every 1.0 mL of TRIzol™ Reagent used, 0.2 mL of chloroform (Millipore Sigma, St. Louis, MO, USA) was added. The capped sample was vigorously shaken for 30 s. Following a 3 min room temperature incubation, the sample was centrifuged at 14,000 rpm for 15 min at 4 °C. The aqueous layer was removed and placed in a new 1 mL tube. For every 1.0 mL of TRIzol™ Reagent that was used for homogenization, 0.5 mL of 100% isopropanol (Millipore Sigma, St. Louis, MO, USA) was added to the aqueous layer. The sample was vortexed briefly. Following a 10 min incubation at room temperature, the sample was centrifuged at 14,000 rpm for 15 min at 4 °C. The supernatant was removed and the RNA pellet was washed with 1 mL of 75% ethanol (Millipore Sigma, St. Louis, MO, USA). The sample was briefly vortexed and then centrifuged at 14,000 rpm for 15 min at 4 °C. The wash was discarded. The RNA pellet was allowed to air dry for 5–10 min. The RNA pellet was gently resuspended in 50 μL of Nuclease-Free Water.

The isolated RNA was purified using the RNA Clean and Concentrator kit (Zymo Research, Irvine, CA, USA) per the manufacturer’s instructions, including the in-column DNase I treatment. The concentration of the RNA was determined using the Qubit RNA BR Assay kit (ThermoFisher Scientific, Waltham, MA, USA). Yields ranged from 500 ng–2 μg. The quality of the RNA was determined using the RNA 6000 Nano kit (Agilent Technologies, Santa Clara, CA, USA). The RNA samples had an RNA Integrity Number (RIN) of 4 or higher.

Small RNA libraries were generated from 1 μg inputs of purified RNA using the NEBNext^®^ Multiplex Small RNA Library Prep Set for Illumina (New England BioLabs, Ipswich, MA, USA) following the manufacturer’s protocol. During PCR amplification, a unique index was added to each sample. Each amplified sample was purified using a QIAQuick PCR Purification column (QIAGEN, Hilden, Germany). The purified DNA was eluted from the column in 32 μL of Nuclease-Free Water. The concentration of the purified DNA was measured using the Qubit dsDNA HS assay kit (ThermoFisher Scientific, Waltham, MA, USA). The size distribution was determined by running an aliquot of each library on the Agilent Bioanalyzer High Sensitivity DNA Analysis assay (Agilent Technologies, Santa Clara, CA, USA). The remaining library sample was then size-selected on a Blue Pippin 3% Agarose Gel Cassette, dye-free, with internal standards (Sage Science, Beverly, MA, USA) to enrich for library fragments 17–200 nt in length. The concentration of the enriched fragments was determined using the Qubit dsDNA HS assay kit (ThermoFisher Scientific, Waltham, MA, USA). The fragment size distribution was verified by running an aliquot of the enriched fragments library on the Agilent Bioanalyzer High Sensitivity DNA Analysis assay (Agilent Technologies, Santa Clara, CA, USA).

## 5. Conclusions

In this article, we reported the design and implementation of a novel analysis tool, DANSR, that is able to analyze reads of various lengths to monitor diverse classes of small noncoding RNAs (17–200 nt). As proof of concept, we demonstrated how DANSR can reveal the landscape of known and novel small noncoding RNAs relevant throughout metastatic colorectal cancer progression. However, our optimized tool for small noncoding RNA can be applied broadly to any small RNA sequence data in human development or disease.

## Data Availability

Full source code, installation instructions, examples of running DANSR, and the link to the Docker image are freely available from https://github.com/ChrisMaherLab/DANSR, accessed on 25 October 2021.

## Supplementary Materials

The following are available online at https://www.mdpi.com/article/10.3390/ncrna8010009/s1, Figure S1. Diversity and functions of small noncoding RNA species. Figure S2. DANSR’s heuristic algorithm in optimizing small RNA boundary prediction. Figure S3. Detailed DANSR decision tree model to identify annotated and novel small RNAs. Figure S4. Accurate detection of small RNA read clusters overlapping protein-coding exons. Figure S5. Discovery of novel mature miRNAs with associated hairpin precursors in deep-sequenced CRC samples. Table S1. Examples of existing small RNA analysis tools. Table S2. Institutional colon cancer cohort with matched normal and metastatic samples. Table S3. Small noncoding RNAs discovered and quantified in colorectal cancer samples. Table S4. Differentially expressed small noncoding RNAs in colorectal cancer.
