# Supplementary material for: DANSR: A Tool for the Detection of Annotated and Novel Small RNAs
Source: Noncoding RNA. 2022 Jan 13;8(1):9. doi: 10.3390/ncrna8010009 (PMC8788476; doi:10.3390/ncrna8010009)
Supplement: Supplementary file 1 [file ncrna-08-00009-s001.zip › ncrna-1458322-supplementary-PUB.pdf]

Additional files

Figure S1. Diversity and functions of small noncoding RNA species.

Figure S2. DANSR's heuristic algorithm in optimizing small RNA boundary prediction.

Figure S3. Detailed DANSR decision tree model to identify annotated and novel small RNAs.

Figure S4. Accurate detection of small RNA read clusters overlapping protein-coding exons.

Figure S5. Discovery of novel mature miRNAs with associated hairpin precursors in deep-sequenced CRC samples.

Table S1. Examples of existing small RNA analysis tools.

Table S2. Institutional colon cancer cohort with matched normal and metastatic samples.

Table S3. Small noncoding RNAs discovered and quantified in colorectal cancer samples.

Table S4. Differentially expressed small noncoding RNAs in colorectal cancer.

Figure **S1. Diversity and functions of small noncoding RNA species.** In the full-length range (17-200nt), a diverse range of small noncoding RNA species have been shown to contribute to human development and disease, including both microRNA (<35nt) and mid-sized small noncoding RNAs (36-200nt). In contrast, long noncoding RNAs (lncRNA) are >200nt in length.

17-35nt	36-200nt	>200nt
**microRNA** (miRNA)<br>17-22nt<br>Regulation of gene expression<br>**piwi-interacting RNA** (piRNA)<br>25-33nt<br>Regulation of retro transposons	**small nucleolar RNA** (snoRNA)<br>60-200nt<br>Guiding chemical modifications of other RNAs<br>**transfer RNA** (tRNA)<br>70-90nt<br>Translation of mRNA to protein<br>**small nuclear RNA** (snRNA)<br>100-200nt<br>Processing of pre-messenger RNA<br>**ribosomal RNA** (rRNA)<br>120-160nt<br>Essential for protein synthesis<br>…	**long non-coding RNA** (lncRNA)<br>>200nt<br>Autonomously transcribed RNA that does not encode a protein

Figure S2. DANSR's heuristic algorithm in optimizing small RNA boundary prediction. (A) Small RNA sequencing reads are assigned a weight calculated based on supporting overlapping reads, and low-weight reads are excluded to optimize small noncoding RNA boundary discovery. **(B)-(D)** Executing our algorithm shows significant improvements in boundary discovery for small RNAs with various length ranges.

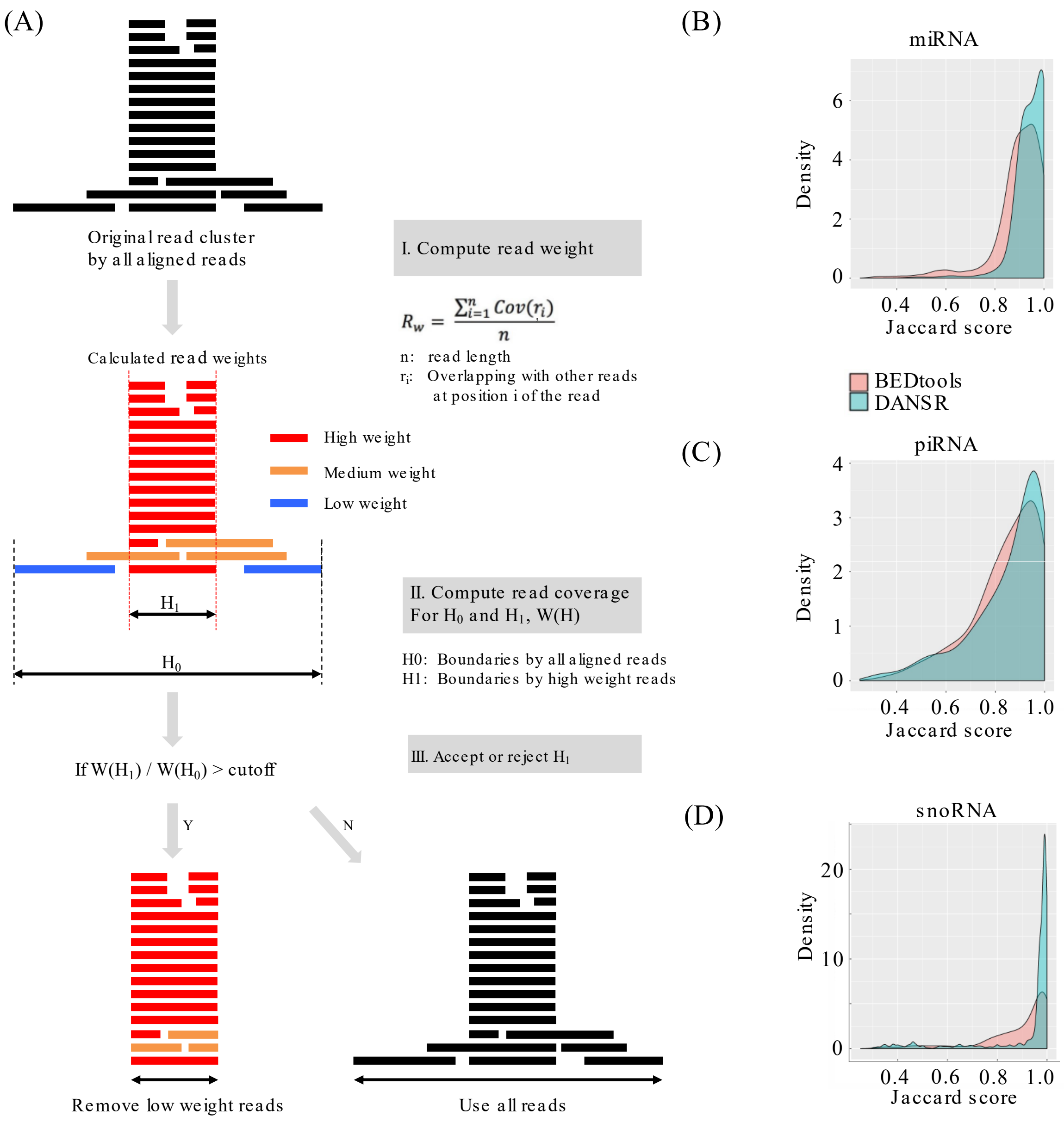

Figure S3. Detailed DANSR decision tree model to identify annotated and novel small RNAs.

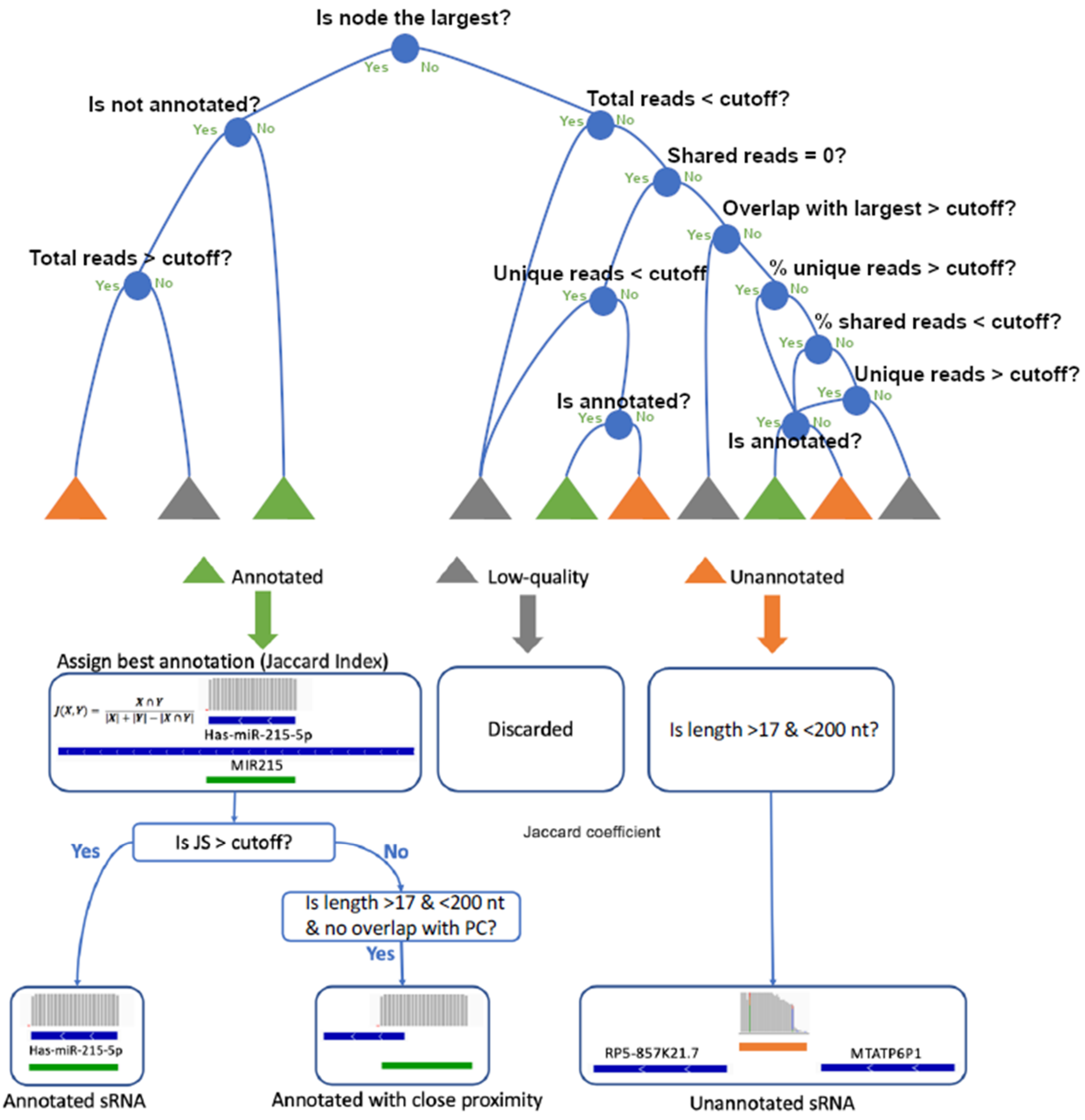

Figure S4. Accurate detection of small RNA read clusters overlapping protein-coding exons. While tools such as Manatee report expression from many protein-coding exons as novel small RNAs, DANSR's boundary optimization and decision tree algorithms ensure accurate reporting of small RNA clusters which overlap protein-coding exons. **(A)** A known snoRNA that overlaps TPT1; **(B)** a known miRNA that overlaps GNAI3.

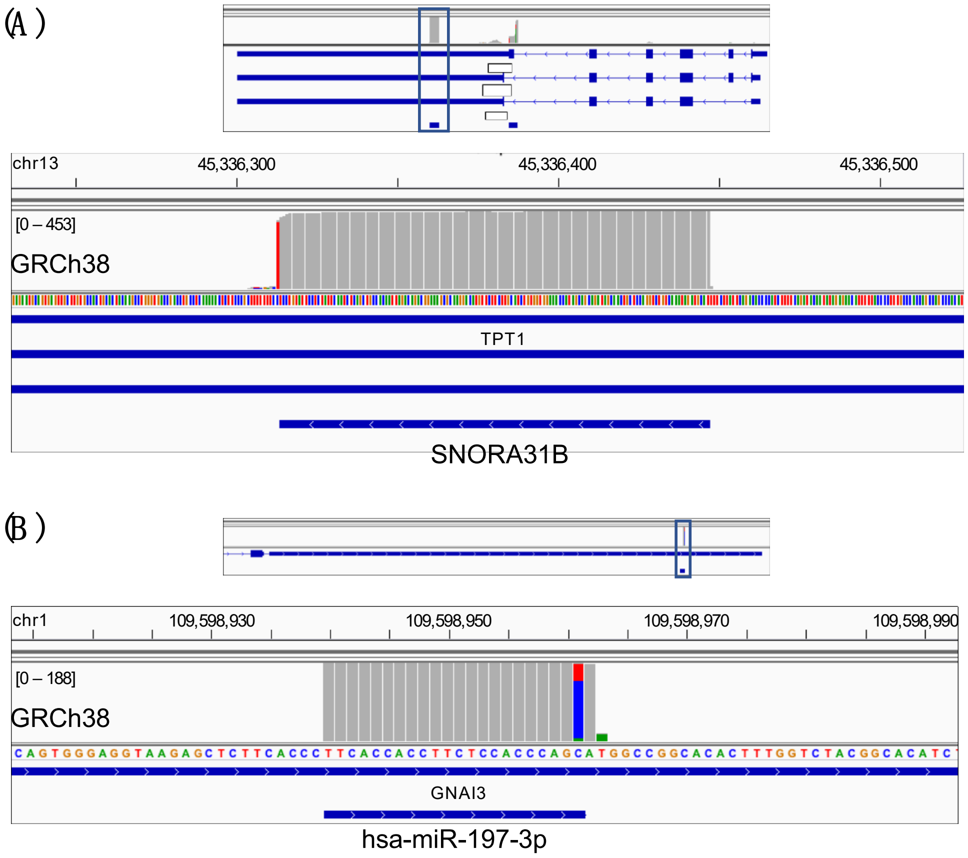

Figure **S5. Discovery of novel mature miRNAs with associated hairpin precursors in deep-sequenced CRC samples. (A)** Novel miRNA (length 23) with its predicted hairpin structure based on up to 40 additional nucleotides extracted in the 3' direction of the remaining portion of the miRNA; **(B)** Novel miRNA (length 24) with predicted hairpin structure in the 5' direction.

(A)

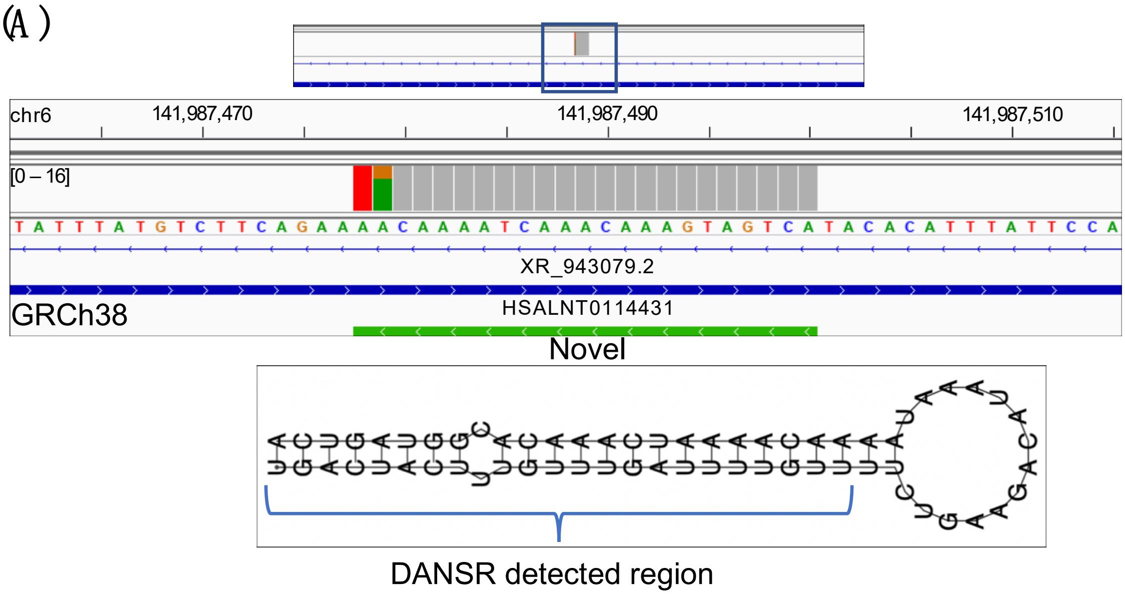

(B)

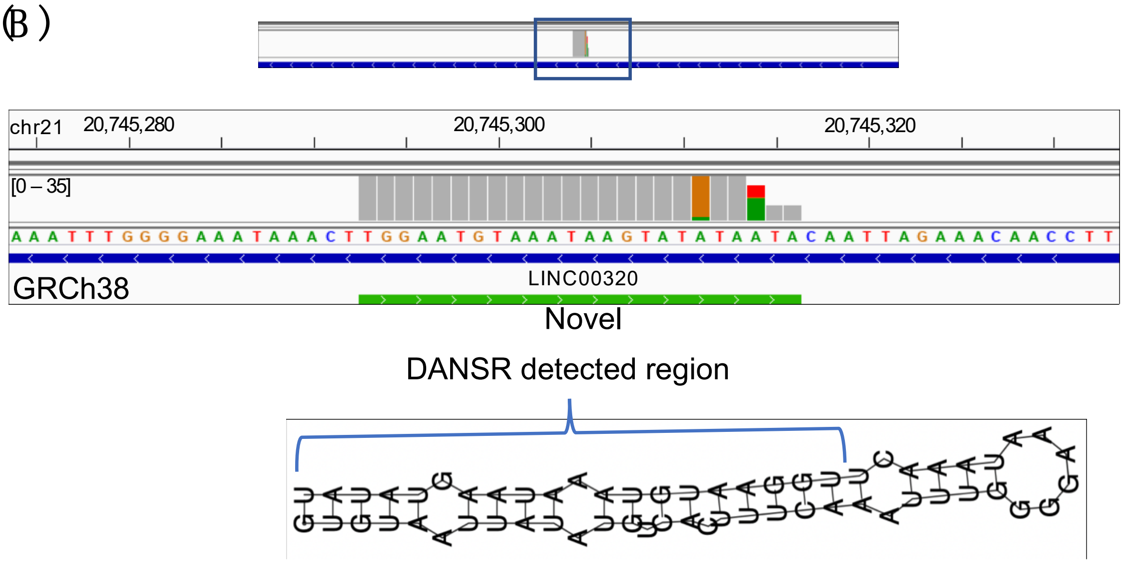

Table S1. Limitations of existing small RNA analysis tools. Existing small RNA analysis tools optimized for processing short sequencing reads (17-35 nucleotides) to monitor microRNA expression, are not suitable to analyze deep sequencing data with various read lengths. Six representing existing tools and their limitations are summarized in the table. On the other hand, DANSR is optimized and validated to analyze deep sequencing data with various read lengths.

Tool	Publication	Problem
Manatee	Handzlik et al, SciRep 2020	Mislabeling / low quality clusters, no boundary prediction
sRNAtoolbox	Rueda et al, NAR 2015	miRNA only
ShortStack	Axtell, RNA 2013	Limited filtering, no annotation
mirTools 2.0	Wu et al, RNA Bio 2013	FASTA: maximum size 30 MB
NorahDesk	Ragan et al, NAR 2012	miRNA and piwiRNA only
DARIO	Fasold et al, NAR 2011	Restricts read length to < 55nt

Table S2. Institutional colon cancer cohort with matched normal and metastatic samples.

Please refer to Table S2.xlsx.

Table S3. Small noncoding RNAs discovered and quantified in colorectal cancer samples.

Please refer to Table S3.xlsx.

Table S4. Differentially expressed small noncoding RNAs in colorectal cancer.

Please refer to Table S4.xlsx.
